# Analytical verification of the Dymind D7-CRP automated analyser

**DOI:** 10.11613/BM.2023.020703

**Published:** 2023-06-15

**Authors:** Merima Čolić, Bojana Magdić, Monika Kolundžić, Jasna Leniček Krleža

**Affiliations:** Department of Laboratory Diagnostics, Children’s Hospital Zagreb, Zagreb, Croatia

**Keywords:** blood cell count, C-reactive protein, haematology, verification

## Abstract

**Introduction:**

The aim of this study was to perform a verification of the Dymind D7-CRP automated analyser and compare it with established analysers.

**Materials and methods:**

Analytical verification included estimation of repeatability, between run precision, within-laboratory precision, and bias in control samples with low, normal and high levels. The acceptance criteria for analytical verification were defined using the European Federation of Clinical Chemistry and Laboratory Medicine (EFLM) 2019 Biological Variation Database. Method comparison between the Dymind D7-CRP and the Sysmex XN1000 for haematological parameters and the Dymind D7-CRP and the Beckman Coulter AU680 for CRP values was performed on 40 patient samples.

**Results:**

Analytical verification criteria were adequately met with the exception of monocyte count for repeatability and within-laboratory precision (13.4% and 11.5%, respectively, acceptance criteria 10.1%) and measurement uncertainty (23.0, acceptance criteria 20.0%) at low level, eosinophil count for BIAS at the low level (37.7%, acceptance criteria 25.2%), basophil count (BAS) for BIAS at the high level (14.2%, acceptance criteria 10.9%), and mean platelet volume (MPV) for repeatability (4.2% and 6.8%), between run precision (2.2% and 4.7%), within-laboratory precision (4.0% and 7.3%) (acceptance criteria 1.7%), and measurement uncertainty (8.0 and 14.6%, acceptance criteria 3.4%) at both the low and high concentrations. Method comparison showed no clinically significant constant or proportional differences for all parameters except BAS and MPV.

**Conclusion:**

The analytical verification of the Dymind D7-CRP showed adequate analytical characteristics. The Dymind D7-CRP can be used interchangeably with the Sysmex XN-1000 for all parameters tested, except BAS and MPV, and with the Beckman Coulter AU-680 for the determination of CRP.

## Introduction

C-reactive protein (CRP) concentration, white blood cell count (WBC), and leukocyte differentiation (WBC DIFF) are the most commonly requested parameters in pediatric emergency departments (ED) on admission of patients with or without fever (*1*). Clinicians rely on rapid turnaround time (TAT) to achieve early diagnosis and treatment of their patients and to allow early discharge of patients from EDs or inpatient facilities (*2*).

According to current published data, in a core laboratory, CRP results from serum samples are available approximately 60-90 minutes after blood is drawn from the patient (*3*). To obtain the serum CRP value, the fully coagulated blood must be centrifuged for approximately 15 minutes, whereas results from whole blood samples are available more quickly because the pre-analytical sample preparation for whole blood samples is shorter (*4*).

In the pediatric population, capillary sampling is important to avoid the effects of total blood volume reduction and is generally less invasive than venipuncture (*5*).

The laboratory department of the Children’s Hospital Zagreb recognized the need to introduce an analyser for simultaneous analysis of blood count (CBC) and CRP from whole blood samples.

The aim of this study was to evaluate the Dymind D7-CRP (Shenzhen Dymind Biotechnology Co., Shenzhen, China), an analyser for the determination of CRP and CBC in whole blood samples, in comparison with standard laboratory tests for these parameters in pediatric patients. To our knowledge, none of the studies published to date have performed verification of the Dymind D7-CRP prior to its use in routine practice.

## Materials and methods

### *S*tudy design

This study was conducted in December 2021 at the Department of Medical Laboratory Diagnostics, Children’s Hospital Zagreb (Zagreb, Croatia). Verification of the Dymind D7-CRP was performed according to the internally developed verification protocol based on the Recommendations of the Croatian Society of Medical Biochemistry and Laboratory Medicine (CSMBLM) and the Croatian Chamber of Medical Biochemists (CCMB) Joint Working Group on Measurement Uncertainty and the Clinical and Laboratory Standards Institute guidelines EP09c: Measurement Procedure Comparison and Bias Estimation Using Patient Samples, 3rd Edition (*6*, *7*).

### Materials

Verification data were collected by analysing commercial control samples in triplicate for five consecutive days. Control samples were analysed at low, normal, and high concentrations. The control samples for haematological parameters were CBC-DH LOT DH2111 (R&D Systems, Minneapolis, USA) at three levels (low, normal, and high). The control samples for CRP concentration were CRP LOT 2021020501 (low and normal concentration values) and CRP LOT 2021122301 (high concentration value) (Shenzhen Dymind Biotechnology Co., Shenzhen, China).

Samples for method comparison were residual capillary and venous samples from pediatric patients collected in December 2021 at the Department of Medical Laboratory Diagnostics, Children’s Hospital Zagreb (Zagreb, Croatia). Samples for CBC and CRP analysis were collected in test tubes with spray-coated K2EDTA (for capillary blood samples Greiner Bio-One GmbH, Kremsmünster, Austria, and for venous samples KABE LABORTECHNIK GmbH, Nümbrecht-Elsenroth, Germany). Measurements on the analysers were performed no later than 2 hours after sample collection. Blood samples were stored at room temperature until the time of analysis. Clotted samples and samples with insufficient volume were excluded from the study. Forty samples were specifically selected to cover the full measurement range of the Dymind D7-CRP. The measurement range for CRP concentration measurement was 0.2-250 mg/L.

The study had the approval of the hospital Ethics Committee.

### Methods

The Dymind D7-CRP is an automated analyser that provides quantitative analytical results for blood count, 5-part white blood cell classification, haemoglobin concentration (HGB) measurement, and CRP concentration in venous and capillary whole blood samples as well as prediluted whole blood samples. The analyser determines CBC parameters using the impedance method, WBC DIFF using semiconductor laser-based flow cytometry, HGB using a colorimetric method, and CRP concentration using immunoturbidimetry.

The present Sysmex XN1000 automated reference haematology analyser (Sysmex Corporation, Kobe, Japan) was used for method comparison of haematology parameters. The measurement methods are the same as those of the Dymind D7-CRP.

The Beckman Coulter AU680 Chemistry Analyser (Beckman Coulter, Brea, USA), an automated biochemistry analyser, was used for method comparison of CRP concentration measurement. It uses latex immunoturbidimetry to determine CRP concentration in serum and plasma samples.

To evaluate analytical performance and clinical applicability, a verification of the analyser and a comparison of methods were performed.

Analytical verification included estimation of repeatability, between run precision, within-laboratory precision, bias, and measurement uncertainty. The acceptance criteria for analytical verification data were defined from the European Federation of Clinical Chemistry and Laboratory Medicine (EFLM) 2019 Biological Variation Database (*8*).

Haematological parameters calculated from directly measured parameters (haematocrit, mean corpuscular haemoglobin (MCH), mean corpuscular haemoglobin concentration (MCHC), coefficient of variation of red cell distribution width (RDW-CV), and leukocyte subpopulation ratio) were excluded from precision studies.

Bias was calculated as a deviation of grand mean (mean value of all mean values of each day for each control sample) from the target value of a specific parameter derived from control sample and declared by the manufacturer. The equation: Bias (%) = [(Grand mean value – Target value)/Target value] x 100.

Method comparison between the Dymind D7-CRP and the Sysmex XN1000 for haematological parameters, and the Dymind D7-CRP and the Beckman Coulter AU680 for CRP values was performed on 40 patient samples.

### Statistical analysis

The age of the patients was expressed as the median (range). Statistical analysis of the method comparison included Bland-Altman deviation analysis and a Passing-Bablok regression analysis. For the Bland-Altman deviation analysis, two separate analyses were performed, one to determine the presence of a constant difference and one to determine the presence of a proportional statistically significant difference between the two instruments. When 95% of the differences are between ± 1.96 times the standard deviation (SD) of the differences, the methods can be used interchangeably. Furthermore, the mean presented with its 95% confidence intervals is considered statistically significant if both those limits are higher or lower than 0.

For the Passing Bablok regression analysis, if the 95% confidence interval for the intercept includes zero as value, there is no systematic error between the two instruments. If the 95% confidence interval of the slope includes one as value, there is no proportional difference between the two instruments.

Statistically significant differences were compared to clinically relevant criteria in order to evaluate if these differences are clinically relevant. Per the Department’s internally developed verification protocol for method verification, the criteria were defined from the Croatian Centre for Quality Assessment in Laboratory Medicine (CROQALM) recommendations for the maximum allowable mean difference (*9*).

Statistical analyses were performed using MedCalc Statistical Software version 19.5.3 (MedCalc Software Ltd, Ostend, Belgium).

## Results

### Analytical verification

During the verification process, all measurement results of the control samples were within their target intervals. Measurement uncertainty, repeatability values, between run precision, within-laboratory precision, and bias for the tested parameters are listed in Table 1.

**Table 1:** Results for repeatability, between run precision, within-laboratory precision, inaccuracy (BIAS) and measurement uncertainty using Dymind D7-CRP automated analyser

**Parameter**	**Control samples**	**Repeatability** **(%)**	**Between run precision** **(%)**	**Within-** **laboratory precision** **(%)**	**EFLM quality requirements** **for imprecision (%)**	**BIAS (%)**	**EFLM quality requirements for BIAS (%)**	**Measurement uncertainty**	**EFLM quality requirements for maximum allowable measurement uncertainty** **(%)**
	L (3.40)	1.6	1.5	2.0	8.1	0.3	7.4	4.0	16.2
WBC (x10^9^/L)	N (8.02)	1.6	1.2	1.8	8.1	0.2	7.4	3.6	16.2
	H (18.49)	1.4	0.7	1.3	8.1	0.6	7.4	2.6	16.2
	L (1.67)	2.5	2.7	3.4	10.5	0.0	10.3	6.8	21.0
NEU (x10^9^/L)	N (4.48)	2.1	1.5	2.2	10.5	0.3	10.3	4.4	21.0
	H (11.61)	1.9	1.1	1.9	10.5	2.4	10.3	3.8	21.0
	L (1.34)	2.9	1.4	2.7	8.1	6.7	9.4	5.4	16.2
LYM (x10^9^/L)	N (2.42)	1.7	2.0	2.5	8.1	3.5	9.4	5.0	16.2
	H (3.94)	2.7	2.0	3.0	8.1	5.5	9.4	6.0	16.2
	L (0.22)	13.4	3.4	11.5	10.1	7.3	9.7	23.0	20.0
MON (x10^9^/L)	N (0.55)	6.4	3.6	6.4	10.1	4.7	9.7	12.8	20.0
	H (1.24)	3.8	2.5	3.9	10.1	3.8	9.7	7.8	20.0
	L (0.17)	9.0	7.8	10.7	11.3	37.7	25.2	21.4	22.5
EOS (x10^9^/L)	N (0.57)	7.3	3.8	7.1	11.3	19.3	25.2	14.2	22.5
	H (1.70)	4.4	2.0	4.1	11.3	23.3	25.2	8.2	22.5
	L (2.12)	2.5	2.2	3.0	9.3	0.3	10.9	6.0	18.6
BAS (x10^9^/L)	N (5.82)	1.7	1.0	1.7	9.3	0.8	10.9	3.4	18.6
	H (13.00)	1.5	0.4	1.3	9.3	14.2	10.9	2.6	18.6
	L (2.27)	1.0	0.6	1.0	2.0	2.1	2.6	2.0	3.9
RBC (x10^12^/L)	N (4.56)	0.6	0.3	0.6	2.0	1.5	2.6	1.2	3.9
	H (5.32)	1.2	0.3	1.0	2.0	2.2	2.6	2.0	3.9
	L (57)	1.0	0.8	1.2	2.0	0.0	2.4	2.4	4.1
Hb (g/L)	N (131)	0.7	0.5	0.8	2.0	0.6	2.4	1.6	4.1
	H (168)	0.6	0.4	0.6	2.0	0.4	2.4	1.2	4.1
	L (80.2)	0.1	0.1	0.2	0.6	0.9	1.4	0.4	1.2
MCV (fL)	N (88.1)	0.2	0.2	0.3	0.6	0.8	1.4	0.6	1.2
	H (95.1)	0.1	0.2	0.2	0.6	0.9	1.4	0.4	1.2
	L (56)	5.6	2.3	5.1	5.7	3.5	7.6	10.2	11.4
PLT (x10^9^/L)	N (256)	2.2	0.18	1.8	5.7	5.5	7.6	3.6	11.4
	H (511)	1.7	2.0	2.4	5.7	6.8	7.6	4.8	11.4
	L (11.2)	4.2	2.2	4.0	1.7	3.0	2.8	8.0	3.4
MPV (fL)	N (10.1)	0.8	0.8	1.0	1.7	2.6	2.8	2.0	3.4
	H (10.8)	6.8	4.7	7.3	1.7	1.6	2.8	14.6	3.4
	L (4.4)	3.7	3.8	4.9	25.6	5.8	33.9	9.8	51.2
CRP (mg/L)	N (18.5)	1.8	2.1	2.5	25.6	7.4	33.9	5.0	51.2
	H (115)	3.7	1.5	3.4	25.6	18.7	33.9	6.8	51.2
The values of the control samples are specified by manufacturer. L - low level of control sample. N - normal level of control sample. H - high level of control sample. EFLM - European Federation of Clinical Chemistry and Laboratory Medicine. WBC - white blood cell count. NEU - neutrophil count. LYM - lymphocyte count . MON - monocyte count. EOS - eosinophil count. BAS - basophil count. RBC – red blood cells. Hb – haemoglobin. MCV – mean cell volume. PLT – platelets. MPV - mean platelet volume . CRP - C-reactive protein.									

The following parameters, determined as part of the verification protocol using the Dymind D7 CRP analyser, fully met the criteria for analytical verification at all three concentration levels (low, normal, high): WBC, neutrophil count (NEU), lymphocyte count (LYM), RBC, HGB, mean cell volume (MCV), platelets (PLT) and CRP.

Monocyte count (MON), eosinophil count (EOS), basophil count (BAS), and mean platelet volume (MPV) partially met the criteria. The criteria for measurement uncertainty, repeatability, between run precision, within-laboratory precision, and BIAS were met for all parameters at normal concentrations.

### Method comparison

The median age of patients whose blood samples were used for method comparison was 7 years (range: 3 weeks-17 years). There were 28 (0.70) capillary blood samples, and 12 (0.30) venous blood samples.

**Figure 1:**
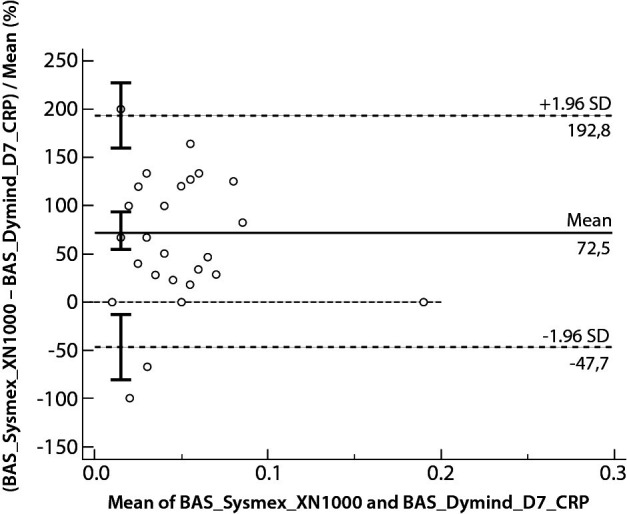
Bland-Altman graph of proportional difference for basophil count (BAS). Solid horizontal lines represent the mean difference between BAS measures on two analysers, the dashed lines represent the mean difference ± 1.96 times the standard deviation, and the solid vertical lines represent their 95% confidence intervals.

**Figure 2:**
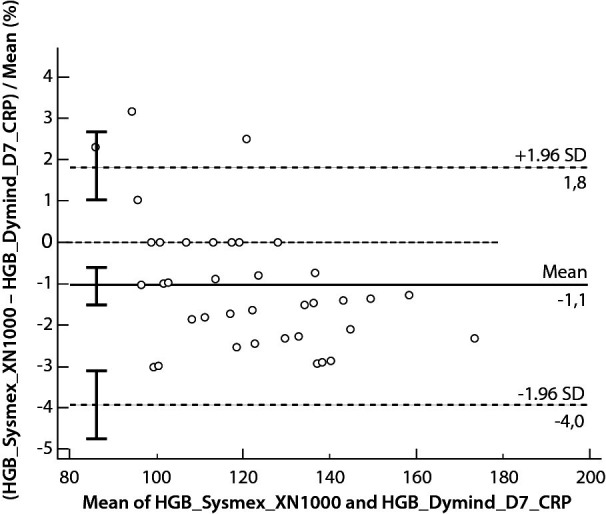
Bland-Altman graph of proportional difference for haemoglobin (Hb). Solid horizontal lines represent the mean difference between HB measures on two analysers, the dashed lines represent the mean difference ± 1.96 times the standard deviation, and the solid vertical lines represent their 95% confidence intervals.

**Figure 3:**
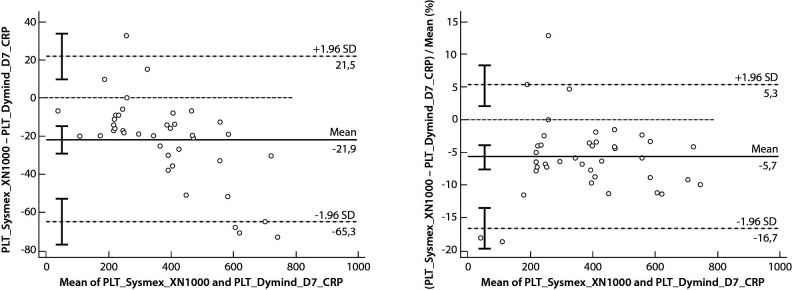
Bland-Altman graph of constant and proportional difference for platelets (PLT). Solid horizontal lines represent the mean difference between PLT measures on two analysers, the dash lines represent the mean difference ± 1.96 times the standard deviation, and the solid vertical lines represent their 95% confidence intervals.

Bland-Altman plots show a scatter plot of differences plotted against the means of two measurements. The analysis shows that WBC, NEU, LYM, MON, EOS, RBC, MCV, MPV, and CRP have no proportional or constant differences between the compared instruments (data not presented). The proportional differences for BAS and HB are shown in Figures 1 and 2, respectively. Figure 3 shows the constant and proportional difference for PLT.

Passing-Bablok regression equations and their 95% confidence intervals (CI) are shown in Table 2. The analysis shows that WBC, NEU, LYM, EOS, BAS, RBC, MCV, and CRP have no proportional or constant differences between the compared instruments. For HB and MPV both proportional and constant differences were found, while MON and PLT have only proportional differences.

**Table 2:** Passing-Bablok regression analysis for the method comparison between the Dymind D7-CRP and the Sysmex XN1000 for haematological parameters, and the Dymind D7-CRP and the Beckman Coulter AU680 for CRP

**Parameter**	**Range tested**	**Regression equation**	**95% CI for slope**	**95% CI for intercept**
WBC (x10^9^/L)	2.66-37.74	y = - 0.12 + 1.00x	0.98 to 1.02	- 0.25 to 0.05
NEU (x10^9^/L)	1.38-25.16	y = - 0.06 + 1.02x	0.99 to 1.03	- 0.13 to 0.03
LYM (x10^9^/L)	0.31-8.63	y = 0.04 + 1.00x	0.97 to 1.02	- 0.03 to 0.09
MON (x10^9^/L)	0.1-4.42	y = 0.00 + 0.83x	0.79 to 0.86	- 0.03 to 0.04
EOS (x10^9^/L)	0.00-1.28	y = 0.01 + 0.96x	0.92 to 1.00	0.00 to 0.01
BAS (x10^9^/L)	0.01-0.19	y = - 0.01 + 0.50x	0.25 to 1.00	- 0.02 to 0.01
RBC (x10^12^/L)	2.93-5.81	y = 0.06 + 1.00x	0.96 to 1.03	- 0.09 to 0.18
Hb (g/L)	87-171	y = - 4.65 + 1.05x	1.02 to 1.08	- 7.88 to - 0.72
MCV (fL)	69.1-109.2	y = 2.24 + 0.96x	0.86 to 1.05	- 5.15 to 10.93
PLT (x10^9^/L)	35-706	y = - 3.11 + 1.07x	1.03 to 1.12	- 16.59 to 8.20
MPV (fL)	8.3-11.1	y = - 4.22 + 1.43x	1.11 to 1.73	- 7.14 to -1.38
CRP (mg/L)	0.9-256.4	y = - 0.75 + 1.03x	0.98 to 1.07	- 2.81 to 0.70
WBC - white blood cell count. NEU - neutrophil count. LYM - lymphocyte count . MON - monocyte count. EOS - eosinophil count. BAS - basophil count. RBC – red blood cells. Hb – haemoglobin. MCV – mean cell volume. PLT – platelets. MPV - mean platelet volume . CRP - C-reactive protein. 95% CI – 95% confidence intervals.				

When comparing MON, Passing-Bablok analysis showed a proportional, statistically significant difference between the Dymind D7-CRP and Sysmex XN1000 analysers, whereas Bland-Altman analysis showed no differences. When comparing the results of the MON measurement with the CROQALM recommendations for the maximum allowable mean difference based on clinically significant differences, we found that the mean difference for MON was 14%, which was lower than the recommended allowable differences of 30%, meaning there was no clinically significant difference.

For BAS, Bland-Altman analysis showed a proportional statistically significant difference. The mean proportional difference was 73%, which is more than the maximum allowable mean difference of 40%.

For Hb, the Bland-Altman analysis showed a proportional statistically significant difference, and the Passing-Bablok analysis showed both a proportional and a constant difference. When comparing the results of Hb measurements with CROQALM recommendations, we found that the mean difference for Hb was 1%, whereas the recommended allowable differences were 5%, so no clinically significant difference was found.

Passing-Bablok analysis showed a proportional statistically significant difference for PLT, and Bland-Altman analysis showed both a proportional and a constant difference for that analyte. Further comparing the results of PLT measurements with CROQALM recommendations, we found that the mean difference for PLT was 6.0%, whereas the recommended allowable differences were 15%, therefore no clinically significant difference was present.

Passing-Bablok showed that both proportional and constant differences in MPV were present. While the mean difference in MPV was 2.9%, which is below the allowable difference of 10% defined by CROQALM, 5/40 pairs of measurements were higher than the allowable difference, leading us to conclude that there is a clinically significant difference between the two methods.

## Discussion

During the verification process, the criteria for analytical verification were adequately met with few exceptions. Monocyte count met all criteria except repeatability, within-laboratory precision, and measurement uncertainty at the low concentration. Eosinophyl count met all criteria except BIAS at the low concentration, BAS met all criteria except BIAS at the high concentration. Mean platelet volume did not meet the criteria for repeatability, between run precision, within-laboratory precision, and measurement uncertainty at both the low and high concentrations.

Two comparative statistical analyses, Bland-Altman deviation analysis and Passing-Bablok regression analysis, were used for method comparison. The purpose of these analyses is to evaluate the degree of agreement to determine if the methods are interchangeable. They show whether there is a statistically significant difference, but it is important to determine whether there is also a clinically significant difference (*10*, *11*).

Although a statistically significant difference was found between Dymind D7-CRP and Sysmex XN1000 in the measured values of MON, HGB, and PLT, these differences were not considered clinically significant. Of the twelve analytes tested, only two showed clinically significant differences, BAS and MPV.

The finding of clinically significant proportional difference detected for BAS was analogous to the results of Velizarova *et al.*, who compared a similar analyser, the Dymind DH76, with the XN1000, and to those of Lin *et al.*, who compared the Mindray BC-7500 with manual differential counting (*12*, *13*). These differences are to be expected and can be explained by the low number of basophils in the blood.

When interpreting MPV differences, one of the factors that should be considered is that analysis with Dymind D7-CRP was always performed after analysis with the Sysmex XN1000 and that measurement of MPV with the impedance method can be affected by sample interaction with the anticoagulant K2EDTA, which causes platelet swelling and can lead to a 7.9% (or 0.7 fL) increase in size within 30 minutes (*14*).

A limitation of this study is that only samples from pediatric patients were used, so these results should be further tested before being applied to an adult population. Because the samples were specifically selected to test the range of the Dymind D7-CRP analyser, the patients were not evenly selected by age, so the median age of the patient group was 7 years. This selection also resulted in a discrepancy between capillary and venous blood samples with ratios of 0.70 and 0.3, respectively. In addition, the study did not include a comparison of differential white blood cell counts with manual microscopic evaluation.

Although the analytical evaluation of the instrument did not meet the established criteria for all parameters, it met the recommendations based on clinically significant differences, so the differences do not affect clinicians’ decision making. The results of the analytical verification and method comparison support the use of the Dymind D7 analyser in the emergency laboratory for the determination of CBC and CRP. The high demand for rapid determination of these parameters in both hospitals and primary care settings is not surprising, as they can guide patient care. A 2016 study in the pediatric population showed how a CRP < 5 mg/L rules out severe infection and could be used by primary care physicians to avoid unnecessary hospital referrals (*15*). An analyser that can rapidly determine CRP concentration from a whole blood sample could be valuable for patient management.

In conclusion, the analytical verification of the Dymind D7-CRP automated analyser showed adequate analytical characteristics. Based on the analyses of both statistical and clinical differences, we conclude that the Dymind D7-CRP is interchangeable with the XN-1000 for all parameters tested except MPV. For the determination of CRP, the Dymind D7-CRP can be used interchangeably with the Beckman Coulter AU-680.

## Data Availability

The data generated and analysed in the presented study are available from the corresponding author on request.
